# Drug Retention of First-Line Biologic Therapies in Rheumatoid Arthritis: Real-World Evidence From a Moroccan Cohort

**DOI:** 10.7759/cureus.108523

**Published:** 2026-05-08

**Authors:** El Marbouh El Kacem, Abderrahim Majjad, Wissal Belhadj, Hamza Toufik, Laila Taoubane, Ahmed Bezza

**Affiliations:** 1 Rheumatology, Mohammed V Military Training Hospital, Rabat, MAR; 2 Rheumatology, Mohammed V Military Training Hospital, rabat, MAR

**Keywords:** biologic therapy, drug retention, real-world data, rheumatoid arthritis, treatment persistence

## Abstract

Drug retention is a meaningful real-world composite endpoint reflecting both long-term effectiveness and tolerability of biologic therapies in rheumatoid arthritis (RA). Data from North African cohorts remain scarce, and sociocultural determinants of treatment persistence are poorly explored. We conducted a retrospective observational study including RA patients who initiated first-line biologic therapy at a Moroccan tertiary center. Drug retention was defined as the time from initiation to permanent discontinuation for any reason. To compare drug retention across biologic agents, the Kaplan-Meier method was applied alongside the log-rank test. Cox proportional hazards regression was subsequently used to determine baseline factors independently associated with treatment discontinuation.

One hundred and thirty patients met the inclusion criteria, with a clear female predominance (n = 112, 86.2%) and a mean age of 58.7 ± 12.9 years. Rituximab was the most frequently prescribed first-line biologic (n = 72, 56.1%), followed by Etanercept and Tocilizumab (n = 21, 16.2% each). Drug retention differed significantly across biologics (log-rank p = 0.018). Taking Adalimumab as the reference, three biologics showed significantly better retention: Etanercept (hazard ratio (HR) = 0.14, p = 0.001), Rituximab (HR = 0.26, p = 0.006), and Tocilizumab (HR = 0.07, p < 0.001). Additional independent predictors of better retention included concomitant conventional synthetic disease-modifying antirheumatic drug (csDMARD) therapy (HR = 0.39, p = 0.014), lower baseline Simplified Disease Activity Index (SDAI) (HR = 0.96, p = 0.020), and primary education level (HR = 0.32, p = 0.033).

In this real-world Moroccan cohort, non-TNF (tumor necrosis factor) biologics demonstrated favorable retention compared to Adalimumab. Beyond treatment type, both clinical and sociocultural factors were associated with persistence. Given the retrospective observational design and the inherent risk of channeling bias, these results should be interpreted with appropriate caution.

## Introduction

Rheumatoid arthritis (RA) is a chronic systemic autoimmune disorder driven by persistent synovial inflammation and structural joint damage, with numerous extra-articular manifestations that collectively contribute to significant disability and impaired quality of life [1]. Its management relies on early diagnosis and a treat-to-target approach targeting remission or sustained low disease activity [2,3].

Although csDMARDs (conventional synthetic disease-modifying antirheumatic drugs), particularly methotrexate, form the first-line therapeutic backbone in RA, a substantial number of patients ultimately require escalation to b/tsDMARDs (biologic/targeted synthetic disease-modifying antirheumatic drugs), a class that includes TNFi (tumor necrosis factor inhibitor), IL-6Ri, Rituximab, Abatacept, and JAKi (Janus kinase inhibitor) [2]. Despite their proven efficacy, 30%-40% of patients still experience inadequate responses or adverse events, leading to treatment withdrawal [4]. In such cases, switching to another b/tsDMARD is an essential therapeutic option.

Drug retention, defined as the duration a patient continues a prescribed biologic therapy, is increasingly recognized as a meaningful composite endpoint that reflects both long-term efficacy and tolerability in real-world settings. A study by Manfredi et al. indicated that around 50% of RA patients switch from their initial biologic treatment within the third year, with the primary reasons being primary or secondary inadequate responses or adverse events [5]. These findings highlight the importance of identifying predictors of treatment retention in routine clinical practice.

Patterns of biologic drug retention may differ across healthcare settings due to variations in therapeutic access, prescribing practices, and patient profiles, underscoring the need for real-world data from diverse clinical contexts. The primary objective of this study was to evaluate first-line biologic drug retention in a monocentric Moroccan RA cohort. The secondary objective was to identify clinical, demographic, and sociocultural predictors of treatment persistence. Understanding these patterns could inform treatment strategies, improve long-term outcomes, and support the development of personalized therapeutic approaches for RA patients in similar healthcare settings.

## Materials and methods

Study design and patients

This retrospective observational study was conducted at the Rheumatology Department of Mohammed V Military Training Hospital, Rabat, Morocco, and included patients who had initiated at least one biologic therapy between January 2010 and December 2023. Data were collected between January 2024 and February 2026 through systematic review of medical records, outpatient clinic files, and biologic therapy records retrieved during routine consultations for intravenous biologic infusion or prescription renewal, covering the entire follow-up period from biologic initiation until treatment discontinuation or end of follow-up.

Patients

Inclusion criteria comprised a confirmed RA diagnosis per the 2010 ACR/EULAR (American College of Rheumatology/European Alliance of Associations for Rheumatology) classification criteria [6], age ≥ 18 years at the time of inclusion, and initiation of at least one biologic therapy during the study period. Exclusion criteria included incomplete medical records, a concomitant systemic autoimmune disease, or biologic therapy prescribed for an indication other than RA.

Clinical assessment

Baseline sociodemographic and clinical data were systematically recorded for each patient, including age, sex, educational level, and residential setting. Comorbid conditions were recorded, including diabetes mellitus, hypertension, cardiovascular disease, dyslipidemia, thyroid disorders, chronic kidney disease, and smoking status.

RA-specific data included disease duration, diagnostic delay, RF and anti-CCP (anti-cyclic citrullinated peptide) serostatus, radiographic joint erosions, and clinically assessed joint deformities.

Disease activity was measured using the DAS28-CRP (Disease Activity Score in 28 joints using C-reactive protein), CDAI (Clinical Disease Activity Index), and SDAI (Simplified Disease Activity Index) [7-9]. Health-related quality of life and pain intensity were evaluated using the HAQ (Health Assessment Questionnaire) [10] and a 0-10 VAS (visual analog scale), where 0 indicates no pain and 10 the worst imaginable pain, respectively [11].

Biologic therapy data

The following data regarding first-line biologic therapy were collected: the type of biologic agent initiated, the date of initiation, the duration of treatment, the reason for discontinuation, if applicable, and the type of subsequent switch, if performed.

Reasons for discontinuation were classified as follows: primary failure, defined as failure to achieve remission or low disease activity according to EULAR recommendations [2]; secondary failure, defined as loss of response after an initial adequate response [2]; and adverse events, including allergic reactions and other drug-related toxicities.

Outcome definition and statistical analysis

Drug retention was defined as the time elapsed from first-line biologic initiation to permanent discontinuation for any reason, with patients still on their initial biologic at the end of follow-up considered as censored observations.

Normally distributed continuous variables were expressed as mean ± SD, while non-normally distributed variables were reported as median with IQR (interquartile range). Categorical variables were presented as frequencies and percentages.

Drug retention was estimated by the Kaplan-Meier method, and differences across biologic agents were assessed using the log-rank test. Baseline predictors of discontinuation were identified through univariable and multivariable Cox proportional hazards regression. The proportional hazards assumption was formally tested using Schoenfeld residuals prior to final analysis. Treatment allocation was based on physician judgment, taking into account disease severity, comorbidities, patient preferences, drug availability, and cost-effectiveness considerations, which may have introduced channeling bias. Adalimumab was selected as the reference category given its status as the most widely prescribed first-line biologic in international registries, thereby providing the most clinically meaningful comparator. The type of biologic agent was included as a covariate given its established association with drug retention in previous registries. Variables reaching p < 0.20 in univariable analysis and deemed clinically relevant were entered into the multivariable Cox model. Results were reported as hazard ratios (HR) with 95% confidence intervals (CI).

All variables were screened for missing data. Those exceeding a 5% missingness threshold were excluded from multivariable analysis, and the overall rate of missing data for key model variables remained acceptably low.

Statistical analyses were carried out using Jamovi software (version 2.7.4.0, The jamovi Project, Sydney, Australia), with a two-sided p-value ≤ 0.05 set as the threshold for statistical significance.

Ethical considerations

This study complied with the principles of the Declaration of Helsinki, and written informed consent was obtained from all active patients prior to enrollment. For patients included retrospectively who were deceased or lost to follow-up, a waiver of consent was sought, justified by the retrospective nature of the analysis based on routine care data, the practical impossibility of recontacting these patients, and the guarantee of strict anonymization of all collected data. The study protocol received ethical clearance from the Research Ethics Committee of the Faculty of Medicine and Pharmacy of Rabat (approval number: 23/26).

## Results

A total of 130 patients met the inclusion criteria, and their sociodemographic and clinical characteristics are summarized in Table 1. Of the included patients, 116 (89.2%) were seropositive, with a mean disease duration of 16.5 ± 9.44 years and a median diagnostic delay of 1 year (IQR: 1-4). Inflammatory markers were moderately elevated, with a median ESR of 24 mm/h (IQR: 10-39.3) and CRP of 5.6 mg/L (IQR: 1.8-13.2). Disease activity reflected overall low to moderate activity at biologic initiation, with a median DAS28-CRP of 2.92 (IQR: 1.96-4.20) and a median HAQ of 0.7 (IQR: 0.3-1.10), indicating mild functional impairment. Radiographic erosions were present in 105 patients (80.8%), and joint deformities in 44 (33.8%). Concomitant csDMARDs were used in 98 patients (75.4%), and corticosteroids in 23 (17.7%). The median interval between RA diagnosis and biologic therapy initiation was four years (IQR: 1.25-9). 

**Table 1:** Patients characteristics. ^¶^Expressed as number (percentage). ^δ^Expressed as mean±standard deviation. ^§^Expressed as median and interquartile interval. BMI: body mass index, VAS: Visual Analogue Scale, CRP: C-reactive protein, ESR: erythrocyte sedimentation rate, DAS28 CRP: Disease Activity Score 28 CRP, SDAI: Simplified Disease Activity Index, CDAI: Clinical Disease Activity Index, HAQ: Health Assessment Questionnaire, csDMARD: conventional synthetic disease-modifying antirheumatic drug.

Characteristics	Value, N = 130
Age ^δ^	58.7±12.9
Female^¶^	112 (86.2)
Illiterate^¶^	70(53.8)
Primary study^¶^	20(15.4)
Secondary school^¶^	27(20.8)
Higher education^¶^	13(10)
Rural^¶^	33 (25.4)
Urban^¶^	97 (74.6)
BMI^δ^	26.5±4.71
High blood pressure^¶^	35(26.9)
Diabetes^¶^	18(13.8)
Dyslipidemia^¶^	15(11.5)
Dysthyroidism^¶^	13(10)
Smoking^¶^	4(3.1)
Total knee prosthesis^¶^	4(3.1)
Total hip prosthesis^¶^	4(3.1)
History of depression^¶^	8(6.2)
Chronic kidney disease^ ¶^	23(17.8)
Heart disease^¶^	8(6.2)
Pneumococcal vaccination	63(48.5)
RA serologic status^¶^	
Positive	116(89.2)
Negative	14(10.8)
Disease duration^δ^	16.5±9.44
Diagnostic delay (in years)^§^	1(1;4)
VAS^δ^	3.34±1.6
ESR^§^	24(10;39.3)
CRP^§^	5.6(1.8;13.2)
DAS28 CRP^§^	2.92(1.96;4.2)
CDAI^§^	9(4;16)
SDAI^§^	10(4.4;19.4)
HAQ^§^	0.7(0.3;1.10)
Joint deformity^¶^	44(33.8)
Presence of erosions on conventional radiographs^¶^	105(80.8)
CsDMARD use^¶^	98(75.4)
Corticosteroid use^¶^	23(17.7)
Interval between RA diagnosis and biologic therapy initiation^§^	4(1.25;9)

Biologic therapy distribution and drug retention

The most frequently prescribed first-line biologic was Rituximab (n = 72, 56.1%), followed by Etanercept (n = 21, 16.2%), Tocilizumab (n = 21, 16.2%), Adalimumab (n = 10, 7.7%), Infliximab (n = 4, 3.1%), and Golimumab (n = 1, 0.7%).

**Figure 1:**
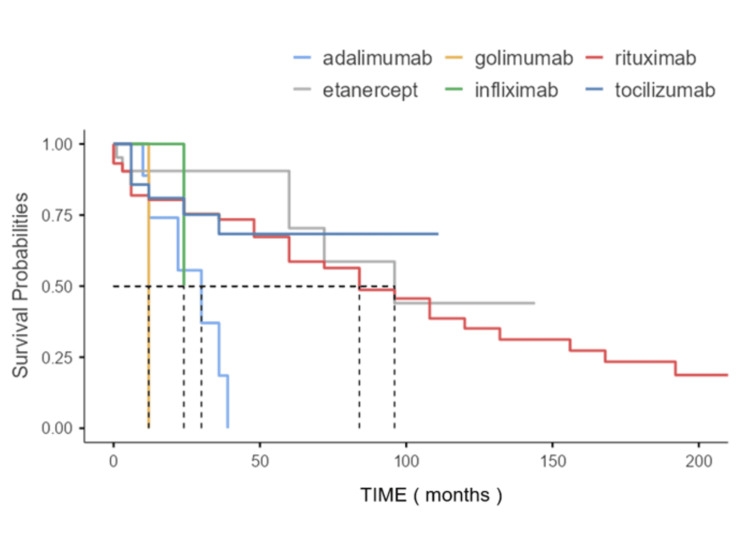
Kaplan-Meier survival analysis of the persistence of different biologic therapies in rheumatoid arthritis. Kaplan-Meier survival curves illustrating drug retention over time for the six first-line biologic therapies. The X-axis represents follow-up time in months, and the Y-axis represents the probability of remaining on the initial biologic therapy. The dashed horizontal line indicates the median survival threshold (0.50). Overall differences in retention rates across biologic agents were statistically significant (log-rank p = 0.018).

Drug retention differed significantly between biologic agents (log-rank p = 0.018). Kaplan-Meier survival curves are illustrated in Figure 1. Median drug survival was longest for Etanercept (96 months) and Rituximab (84 months), followed by Adalimumab (30 months) and Infliximab (24 months). Tocilizumab and Golimumab did not reach their median survival time during the follow-up period, suggesting favorable long-term retention for these agents, albeit based on smaller sample sizes (n = 21 and n = 1, respectively). 

Factors associated with biologic drug discontinuation

The global test for the proportional hazards assumption was significant (p = 0.0008), indicating a non-proportional effect for specific variables, notably the type of biologic agent (p = 0.0001) and concomitant csDMARD use (p = 0.049). For these variables, the reported hazard ratios (HR) represent the average effect over the follow-up period. For all other covariates, the proportional hazards assumption was maintained, indicating a constant effect over time.

In univariable Cox proportional hazards analysis, several factors were examined for their association with biologic drug retention.

Regarding the type of biologic agent, using adalimumab as the reference, Etanercept (HR = 0.21, 95% CI: 0.07-0.68, p = 0.009), Rituximab (HR = 0.34, 95% CI: 0.14-0.85, p = 0.021), and Tocilizumab (HR = 0.21, 95% CI: 0.07-0.68, p = 0.009) were each associated with a significantly lower risk of discontinuation in univariable analysis. Golimumab and Infliximab did not reach statistical significance, likely due to their limited sample sizes (n = 1 and n = 4, respectively).

Regarding education level, using illiteracy as the reference, patients with primary education showed a significantly reduced discontinuation risk (HR = 0.29, 95% CI: 0.10-0.83, p = 0.021), whereas those with higher education exhibited a paradoxically elevated discontinuation risk (HR = 3.15, 95% CI: 1.44-6.86, p = 0.004). No significant association was observed for secondary education (HR = 0.70, p = 0.316).

Concomitant conventional synthetic DMARD use showed a trend toward better retention (HR = 0.57, 95% CI: 0.32-1.02, p = 0.057), as did lower SDAI scores (HR = 0.97, 95% CI: 0.95-1.00, p = 0.057). Age, diagnostic delay, disease duration, presence of erosions, and sex were not significantly associated with drug retention in univariable analysis.

In multivariable Cox proportional hazards analysis, Etanercept (HR = 0.14, 95% CI: 0.04-0.47, p = 0.001), Rituximab (HR = 0.26, 95% CI: 0.10-0.68, p = 0.006), and Tocilizumab (HR = 0.07, 95% CI: 0.02-0.27, p < 0.001) remained independently associated with a significantly lower risk of drug discontinuation compared to Adalimumab. Concomitant conventional synthetic DMARD use (HR = 0.39, 95% CI: 0.19-0.83, p = 0.014) and lower disease activity as measured by SDAI (HR = 0.96, 95% CI: 0.93-0.99, p = 0.020) were also independently associated with better drug retention. Primary education level maintained its protective association (HR = 0.32, 95% CI: 0.11-0.91, p = 0.033), whereas the significant association observed for higher education level in univariable analysis did not persist after adjustment (HR = 1.87, p = 0.197), suggesting confounding by other covariates. The type of biologic agent was included as a covariate given its established association with drug retention in previous registries. 

**Table 2:** Univariate and multivariate analysis of the factors associated with drug discontinuation. Univariable and multivariable Cox proportional hazards regression models were used to identify predictors of biologic drug discontinuation. Results are expressed as HR with 95% CI. A p-value ≤0.05 was considered statistically significant. HR: hazard ratio, CI: confidence interval, csDMARD: conventional synthetic disease-modifying antirheumatic drug, DAS28-CRP: Disease Activity Score in 28 joints with C-reactive protein, SDAI: Simplified Disease Activity Index, SD: standard deviation.

Variables	Categories	HR (Univariable) (95% CI)	p-value	HR (Multivariable) (95% CI)	p-value
Educational level	Illiterate	Reference	–	Reference	–
	Primary	0.29 (0.10–0.83)	0.021	0.32 (0.11–0.91)	0.033
	Secondary	0.70 (0.35–1.39)	0.311	0.55 (0.27–1.13)	0.104
	Higher	3.15 (1.44–6.86)	0.004	1.87 (0.72–4.86)	0.197
Sex	Female	Reference	–	–	–
	Male	0.68 (0.27–1.72)	0.418	–	–
Presence of erosions	Absent (0)	Reference	–	–	–
	Present (1)	2.25 (0.89–5.69)	0.086	–	–
csDMARD therapy	No	Reference	–	Reference	–
	Yes	0.57 (0.32–1.02)	0.057	0.39 (0.19–0.83)	0.014
First biologic	Adalimumab	Reference	–	Reference	–
	Etanercept	0.21 (0.07–0.69)	0.010	0.14 (0.04–0.47)	0.001
	Golimumab	1.95 (0.23–16.29)	0.538	1.84 (0.22–15.48)	0.576
	Infliximab	0.38 (0.05–3.18)	0.374	0.24 (0.03–2.06)	0.195
	Rituximab	0.34 (0.14–0.85)	0.021	0.26 (0.10–0.68)	0.006
	Tocilizumab	0.21 (0.07–0.68)	0.009	0.07 (0.02–0.27)	<0.001
Age	Mean (SD)	0.98 (0.96–1.00)	0.074	-	-
Diagnostic delay	Mean (SD)	0.94 (0.88–1.01)	0.094	-	-
Disease duration	Mean (SD)	1.00 (0.97–1.03)	0.912	-	-
DAS28-CRP	Mean (SD)	0.88 (0.72–1.07)	0.184	-	-
SDAI	Mean (SD)	0.97 (0.95–1.00)	0.057	0.96 (0.93–0.99)	0.020

## Discussion

In this real-world monocentric Moroccan cohort of 130 RA patients, we observed significant heterogeneity in first-line biologic drug retention across biologic agents, with notably long persistence for Etanercept (median 96 months) and Rituximab (median 84 months). Tocilizumab and Golimumab did not reach their median survival time during the follow-up period, suggesting favorable long-term retention, although interpretation is limited by relatively small sample sizes. In contrast, Adalimumab and Infliximab were associated with more rapid discontinuation, with median retention of 30 and 24 months, respectively. Beyond the type of biologic agent, educational level emerged as an independent sociocultural predictor of drug retention, a finding that, to our knowledge, has rarely been reported in the existing literature.

The pattern of superior persistence observed for non-TNF biologics in our cohort is consistent with findings from several international registries. Manfredi et al., analyzing retention rates of biologic and targeted synthetic DMARDs in elderly RA patients from the GISEA (Gruppo Italiano di Studio sulla Early Arthritis) registry, reported that non-TNF biologics were among those with higher retention rates, with primary and secondary inefficacy and infections being the leading causes of withdrawal [5]. Although our population was not exclusively elderly, the overall trend toward better retention for non-TNF agents mirrors their observations.

In the Japanese real-world study by Takami et al., among biologic-naïve RA patients, the retention ranking favored non-TNF inhibitors over TNFi, with Tocilizumab and abatacept demonstrating higher persistence relative to Adalimumab and Infliximab [12]. This pattern closely parallels our observation of superior durability for Tocilizumab and Rituximab compared to Adalimumab and Infliximab.

Furthermore, in the 12-year longitudinal analysis by Murray et al., Etanercept demonstrated a favorable and sustained retention profile among RA patients compared with other biologic agents, and persistence at one year was among the best predictors of long-term continuation [13]. This is consistent with the long median retention of 96 months observed for Etanercept in our cohort. Notably, that study found that most baseline clinical variables did not strongly predict long-term persistence, which resonates with our multivariable finding that age, disease duration, and baseline DAS28-CRP lost significance after adjustment.

The predominance of Rituximab as the most frequently prescribed first-line biologic in our cohort (n = 72, 56.1%) reflects the local prescribing context, where Rituximab is often preferred due to its availability, cost-effectiveness, and established efficacy profile in seropositive RA, which represented (n = 116, 89.2%) of our population. This prescribing pattern differs from Western registries, where TNF inhibitors remain the dominant first-line choice [14,15] and may partly explain the overall favorable retention profile observed in our cohort. However, this non-random treatment allocation introduces potential channeling bias, as treatment selection was likely influenced by patient characteristics, disease severity, and healthcare access. Consequently, while our findings suggest an association between biologic type and drug retention, with Etanercept and Rituximab showing the longest observed median drug survival, these associations should be interpreted with caution. They should not be construed as evidence of causal superiority of one treatment over another, particularly given the observational nature of the study and the inherent risk of channeling bias.

A novel and clinically meaningful finding of our study is the independent association between educational level and biologic drug retention. In multivariable analysis, primary education was associated with a significantly lower risk of discontinuation compared to illiteracy (HR = 0.32, p = 0.033), while higher education was paradoxically associated with increased discontinuation risk in univariable analysis, an association that did not persist after adjustment (HR = 1.87, p = 0.197), suggesting confounding by other covariates. This observation may reflect differences in health literacy, treatment understanding, and adherence to follow-up. However, educational level may also act as a proxy for broader socioeconomic determinants, including access to care and patient engagement. Given the absence of detailed socioeconomic data, residual confounding cannot be excluded, and this finding should be considered hypothesis-generating, warranting confirmation in prospective studies.

Concomitant csDMARD use was independently associated with better drug retention (HR = 0.39, p = 0.014), consistent with current EULAR recommendations advocating combination therapy in RA [2]. This may reflect both pharmacological synergy and improved disease control, which could reduce the risk of secondary failure and subsequent drug discontinuation.

The protective effect of primary education over illiteracy may reflect improved health literacy, better understanding of treatment instructions, and enhanced adherence to follow-up schedules among patients with even a basic level of education. Conversely, the trend toward higher discontinuation among patients with higher education, though not confirmed in multivariable analysis, may reflect greater autonomy in therapeutic decision-making, higher expectations regarding treatment outcomes, or more frequent self-initiated discontinuation. To our knowledge, prior registry-based studies on biologic retention have seldom explored literacy and educational attainment as independent predictors, making this finding particularly relevant in the context of North African and similar healthcare settings where educational disparities remain significant.

Importantly, drug retention represents a composite outcome influenced not only by treatment efficacy and safety but also by multiple non-pharmacological factors, including patient preferences, healthcare accessibility, physician decision-making, and treatment availability. Therefore, retention should not be interpreted as a direct surrogate for pharmacological effectiveness alone.

Limitations

This study has several limitations. First, its retrospective and monocentric design limits generalizability. Second, the considerable imbalance in group sizes across biologic agents, with very small numbers of patients receiving Golimumab (n = 1), Infliximab (n = 4), and Adalimumab (n = 10) compared to Rituximab (n = 72), limits the precision of hazard ratio estimates for these agents and precludes robust comparative interpretation. Furthermore, formal testing of the proportional hazards assumption using Schoenfeld residuals revealed a significant violation for the type of biologic agent (p = 0.0001) and concomitant csDMARD use (p = 0.049), with a significant global test (p = 0.0008). The reported hazard ratios should therefore be interpreted as average estimates over the follow-up period rather than constant effects. Finally, given the observational design and potential channeling bias, the reported hazard ratios should be interpreted with caution and should not be construed as evidence of causal superiority of any biologic agent. The conclusions drawn reflect associations identified in an observational cohort and should not be used to guide treatment selection without confirmation from prospective studies.

Despite these limitations, this study provides original real-world data on biologic drug retention in a North African RA cohort, highlighting both the favorable persistence of non-TNF biologics and the underexplored role of educational level as a predictor of treatment continuity. These findings may inform personalized therapeutic strategies and patient education interventions in similar healthcare contexts.

## Conclusions

This real-world monocentric study provides original evidence on first-line biologic drug retention in a North African RA population. Our findings suggest an association between biologic type and drug retention, with Etanercept and Rituximab showing the longest observed median drug survival and Tocilizumab demonstrating particularly favorable long-term persistence in our cohort; however, these associations should not be interpreted as evidence of causal superiority given the observational design and the potential for channeling bias.

Beyond the type of biologic agent, concomitant csDMARD use, lower baseline disease activity, and educational level independently predicted treatment continuity, highlighting the interplay between clinical and sociocultural determinants of biologic retention. These findings underscore the importance of combination therapy, early therapeutic optimization, and patient education strategies in sustaining biologic treatment in routine clinical practice.

However, these findings should be interpreted in light of the study’s observational design, the non-proportional hazards observed for certain variables, and potential residual confounding. Larger multicenter studies across diverse healthcare settings are warranted to confirm these associations and further refine personalized therapeutic strategies in RA.
